# Plant Oil Nano-Emulsions as a Potential Solution for Pest Control in Sustainable Agriculture

**DOI:** 10.1007/s13744-024-01243-5

**Published:** 2025-01-30

**Authors:** Amany D. Abd-Elnabi, Elham Abdel Fattah El-sawy, Mohamed E. I. Badawy

**Affiliations:** 1 Cotton Leafworm Research Department, Plant Protection Research Institute, Agricultural Research Center, Giza, Egypt; 2 Vegetable and Aromatic Plant Insects Research Department, Plant Protection Research Institute, Agricultural Research Center, Giza, Egypt; 3 Department of Pesticide Chemistry and Technology, Faculty of Agriculture, Alexandria University, 21545-El-Shatby, Alexandria, Egypt

**Keywords:** Plant oil nano-emulsions, Pest control, Sustainable agriculture, Eco-friendly pesticides

## Abstract

The increasing demand for sustainable and eco-friendly pest control methods has led to a growing interest in the development of novel, plant-based pesticides. In this study, we investigated the potential of nano-emulsions containing plant oils (*Portulaca oleracea*, *Raphanus sativus*, and *Rosmarinus officinalis*) as a new approach for controlling three major pests: *Aphis gossypii*, *Spodoptera littoralis*, and *Tetranychus urticae*. Using ultrasonication, we prepared stable and uniform nano-emulsions characterized by thermodynamic properties, dynamic light scattering (DLS), and transmission electron microscopy (TEM). The results showed that the nano-emulsions were effective in controlling the three pests, with the most potent activity observed against *Aphis gossypii*. Our findings suggest that plant oil nano-emulsions have the potential to be used as a sustainable and eco-friendly alternative to traditional pesticides. The use of these nano-emulsions could provide a new approach to manage pest populations, reducing the environmental impact of pesticide use, and promoting sustainable agriculture.

## Introduction

Numerous harmful pests cause economic losses in Egypt such as cotton leafworm, aphids, and mites. The cotton leafworm *Spodoptera littoralis* (Boisduval) (Lepidoptera: Noctuidae) is one of the most significant agricultural pests that feeds on over 80 different crops including important crops such as cotton, corn, peanuts, vegetables, and soybeans. It can cause significant economic damage which make it a major concern for farmers worldwide (Salama et al. 1971; Pineda et al. 2007). Moreover, the sucking pest can cause significant economic loss by affecting a wide range of crops; the cotton aphid *Aphis gossypii* Glover (Hemiptera: Aphididae) causes damage to crops due to the direct feeding which can kill the host, or indirectly through contamination with honeydew and its impact as a plant virus vector (Ebert and Cartwright 1997). Furthermore, *Tetranychus urticae* Koch (Acari: Tetranychidae) can cause significant economic loss by affecting over 3800 host species which include ornamentals, fruits, cotton, and vegetables (Attia et al. 2013).

Pest control needs a large amount of pesticides; the world consumed about 3.70 million tons of active ingredients in 2022 (FAO 2024). However, the misuse of pesticides causes many environmental contamination problems in air, groundwater, and soil. In addition, they cause a decline in populations of beneficial soil microorganisms, soil fertility, and harm non-target organisms (WHO 1990; Gunnell et al. 2007; Dhananjayan 2012; Dhananjayan and Ravichandran 2014; Shao and Zhang 2017; Doolotkeldieva et al. 2018; Woodrow et al. 2018; Ippolito and Fait 2019).

Currently, to reduce the bad influence of pesticides, scientists are searching for new, safe, and biodegradable compounds that can combat pests with fewer side effects on the environment. Thus, green pesticides may be an alternative efficient tool for pest management. Plants are known to produce many phytochemicals (terpenoids, alkaloids, flavonoids, steroids, saponins, and tannins) which are naturally toxic to pests (Stankovic et al. 2020). Plant extracts and essential oils have many useful applications in pest control and proved to be green pesticides (Abbassy et al. 2014; Mossa 2016); it can minimize the application of harmful synthetic pesticides in integrated pest management programs (Guleria and Tiku 2009; Khater 2012). For instance, rosemary oil, *Rosmarinus officinalis* L., which has many active components with pesticidal effects has been widely investigated against different pests (Tak et al. 2017; Hanane et al. 2018 Amiri and Bagheri 2020). Furthermore, radish (*Raphanus sativus*, L.), radish extract, has a high content of organic and active compounds such as hydrocarbons, organic acids, aldehydes, ketones, terpenes, alcohols, and sulfur-containing substances (Selyutina and Gapontseva 2016). It is known for its therapeutic capabilities in all sections of the plant (Ahmad et al. 2012). In addition, it has been used to control pests (Alghamdi 2018; Ibrahim et al. 2020). Purslane (*Portulaca oleracea*, L.) is a green leafy vegetable that is commonly found in tropical and subtropical regions around the world. Purslane is known for its numerous medicinal properties and has been referred to as a “Global Panacea” by Xu et al. (2006). It also has insecticidal activity and has been recognized as a “Chinese indigenous pesticide” by Zhongning (2009), Syed et al. (2010), and Wang et al. (2020).

Developing nano-insecticides is a crucial field of study; it may lead to the creation of substitute pest-control methods and lower the danger of insecticide contamination. Nanotechnology offers great promise to improve agriculture production. Developing nano-pesticide is a crucial field of study; it may lead to increase the effectiveness of agrochemicals by developing advanced formulations that are more effective and targeted, and lower the danger of insecticide contamination. Many research cleared the importance and effectiveness of nano-pesticide formulations on the management of different pests and reduction of the global consumption of synthetic chemicals (Sakulku et al. 2009; Ghosh et al. 2013; Ayoub et al. 2017; Abdelrasoul et al. 2018). Nano-emulsions (emulsions with average droplets size range from 10:200 nm) have recently become important as potential controlled delivery vehicles for pesticides (Wang et al. 2007). Nano-emulsion formulations have gained significant interest due to their ability to solubilize lipophilic active ingredients, increase bioavailability, and promote rapid and efficient penetration and thermodynamically stable system (Chaudhary et al. 2024; Gupta et al. 2024). The essential oil-based nano-emulsions may be considered an effective and valuable formulation for delivery.

So, the objectives of the present study are as follows: analyze the chemical composition of *P. oleracea*, *R. sativus*, and *R. officinalis* oils using gas chromatography-mass spectrometry (GC/MS); formulate oil nano-emulsions using the high-energy emulsification method; and characterize the resulting nano-formulations using dynamic light scattering (DLS) and transmission electron microscopy (TEM). In addition, study the suitability of the selected oils and their nano-emulsion formulations in the laboratory for controlling the *A. gossypii*, *S. littoralis*, and *T. urticae* pests.

So far, as we know, there is no study being done on the preparation and evaluation the biological activity of purslane and radish oil nano-emulsions against the studied pests, despite the possibility that this innovative and environmentally responsible method of pest management could be available.

## Materials and Methods

### Chemicals and Plant Oils

The chemicals that were used in this study included acetone, dimethyl sulfoxide (DMSO), n-hexane, and Tween 80. These chemicals were purchased from Pio-chem Chemicals Company, Egypt. Purslane seed oil (*P. oleracea*) and radish (*R. sativus*) were prepared using a simple two-step extraction process. First, portions of the seeds had been grounded in a coffee grinder for 15 s. Subsequently, a sample of a kilogram of this seed powder was immersed in a solution of 5 L of n-hexane for 24 h at room temperature (25 °C). The mixture was filtered, and the remaining solids were re-extracted with another 5 L of n-hexane. The filtrates from both extractions were mixed and concentrated using a rotary evaporator under a vacuum at 40 °C to eliminate the n-hexane solvent. The resulting oil was weighed and stored at 4 °C for further analysis (Zhao et al. 2017). The steam distillation method utilizing the Clevenger apparatus was employed to extract *R. officinalis* essential oil; briefly, in hydro distillation, we use water or steam for extracting bioactive substances; in Clevenger apparatus, fresh rosemary is boiled in water; the volatile essential oils are vaporized during steam distillation and then condensed upon cooling (Boutekedjiret et al. 2003).

### Preparation of Nano-Emulsions

Preparation of nano-emulsions was achieved by a high-energy method described by Badawy et al. (2017) by using 80 ml of water, 10 ml of essential oil, and 10 ml of Tween 80 at a total mass of 100 ml. First, we mixed the water and Tween 80 using a magnetic stirrer at 2500 rpm for 10 min. Then, oil was added dropwise while maintaining the stirring speed. This stirring continued for an additional 30 min. Finally, the emulsions were subjected to sonication for 20 min at a sonication power of 15 kHz and pulses of 9 cycles per second, using an Ultrasonic Homogenizer (HD 2070 with HF generator (GM 2070), booster horn (SH 213 G) with probe microtip MS 73, Ø 3 mm). To prevent overheating and optimize results during the sonication procedure, it is essential to place the nano-emulsion in ice water path to maintain a consistently controlled temperature environment.

### Characterization of Nano-Emulsions

#### Droplet Size, Zeta Potential, and Polydispersity index

The droplet size, zeta potential, and polydispersity index (PDI) were measured using the Zetasizer Nano instrument (ZS Malvern-UK). Before injection, nano-emulsions were diluted with water to mitigate the impact of multiple scattering at room temperature. The measurements were obtained in triplicate and were presented as the average diameters in nanometers (Sobhani et al. 2015; Su et al. 2017).

#### TEM Analysis

The structure of the nano-emulsions droplet was characterized using TEM, specifically the JEOL JSM-1200EX II model (Peabody, MA, USA), which had a 20-µm aperture and operated at 80 kV. The nano-emulsion (50 µL) was diluted with water, then spread onto a copper grid and allowed to dry naturally at room temperature. TEM images were acquired using a charge-coupled device (CCD) camera.

#### Dynamic viscosity

The viscosity of the nano-emulsion was determined using a Rotary Myr VR 3000 digital viscometer at a temperature of 25 ± 0.5 °C. The viscosity of the formulations was measured directly, without any need to dilute the nano-emulsion before conducting the experiments.

#### Thermodynamic Characterization

The stability of the nano-emulsions was tested under various harsh conditions. These tests included heating/cooling cycles the nano-emulsions were kept at both hot (40 °C) and cold (4 °C) temperatures for 2 days each to see if they changed. In freeze/thaw cycles in this test, the nano-emulsions were alternately frozen (− 21 °C) and thawed (21 °C) for at least 24 h for each temperature. In the centrifugation test, the nano-emulsion were centrifuged at high speeds (5000 rpm) for 30 min to check if they separated or creamed. All tests were done three times (triplicate) to ensure accuracy (Kadhim and Abbas 2015).

#### Gas Chromatography–Mass Spectrometry Analysis of Plant Oils

The chemical compositions of purslane, radish, and rosemary oils were examined using gas chromatography combined with a mass spectrometer, which is a TRACE 1300 GC–MS ISQ (Thermo Scientific). The gas carrier (helium) had a velocity of 39 cm/s and a flow rate of 1 mL/min. The oven temperature began at 45 °C and climbed gradually to 165 °C at a rate of 4 °C per minute. It then further increased to 280 °C at a rate of 15 °C per minute before being held for a “post-run” time. The GC/MS analysis of the plant oils was conducted using a TG-5MS capillary column (30 m in length, 0.25-mm internal diameter, and 0.25-µm film thickness; Thermo Scientific) to determine and categorize their chemical composition. A volume of 1 µL of the oil being tested, which was dissolved in hexane at a ratio of 1:10, was injected into the system at a temperature of 280°C. The injection was done using a split/split-less injector in the split-less mode, with a split ratio of 1:10 and a flow rate of 10 mL per minute. The mass spectra were obtained using electron impact ionization (EI) at an energy of 70 electron volts (eV). The mass spectrometer was operated in scan mode, covering a mass range of 50–600 m/z, with a scanning rate of five scans per second. The scan time for each scan was 1.5 s, and the mass range analyzed was from 40 to 300 amu. The entire duration of the analysis was 52 min. The compounds were identified by comparing their mass spectrum patterns and linear retention indices with authentic references and NIST/Wiley databases (MS libraries) under identical GC/MS conditions, using a homologous sequence of alkanes (C8-C24) (Davies 1990).

### Insects and Bioassay Techniques

Three susceptible pest species; cotton aphid *A. gossypii* Glover, (Hemiptera: Aphididae), two-spotted spider mite *T. uritica* Koch (Acari: Tetranychidae), and *S. littoralis* Boisduval (Lepidoptera: Noctuidae), were used in this study for studying the pesticidal activity of the tested oils, and their nano-emulsion formulations. The pest strains were obtained from a continuously maintained culture in the Plant Protection Research Institute (PPRI), Egypt.

Tow bioassay techniques were used in this study. First, the slide-dipping bioassay technique was used for aphids and mites according to the FAO-recommended method (Stribley et al. 1983; Elbanhawy et al. 2019). A small piece of the double-sided tape was fixed to a glass slide: on the tape, ten fully grown aphids or mites were attached on their dorsal side. Then the slides were immersed in various oil concentrations which dissolved in sterile distilled water containing Tween 80 and DMSO as an emulsifying solution for 10 s. However, the treatment with distilled water containing tween 80 and DMSO was utilized as a control. The insects were kept at a temperature of 25 ± 2 °C and a relative humidity of 70%. The mortality percentage was recorded after 24 h and the insects were supposed to be alive when they exhibited a response when being touched with a delicate brush. Each concentration consisted of three duplicates, with 10 adults per replicate, the corrected mortality (%) was calculated using Abbott’s formula (Abbott 1925).

The second method used is the thin film technique against 4th instar larvae of *S. littoralis*. According to Ascher and Eliyahu (1981), different concentrations of oils and their nano-formulations were prepared in 1 ml acetone, the concentrations were applied to the surface of Petri dishes surface then allowed to evaporate under room temperature; Petri dishes treated with 1 ml of acetone only was used as control. Freshly ten pre-starved molted 4th instar larvae of *S. littoralis* were transferred to the plates, after 6 h of treatment each replicate (five rep.) was provided with a part of fresh clean castor leaf and kept in a growth chamber; larval mortality recorded after 24 h of treatment and Abbott’s formula (Abbott 1925) was used to get correction for natural mortality.

### Statistical Analysis

The statistical analysis was performed using the SPSS software version 21 (IBM 2017). Probit analysis was used to determine the LC_50_ and LC_90_ values for the bioassays, utilizing the log dose–response curves. The 95% confidence interval and standard error were computed to assess the range of LC_50_ and LC_90_ values for the oils in mortality experiments. Abbott (1925) was employed to obtain corrected mortality rates.

## Results

### Chemical composition of the tested oils by gas chromatography-mass spectrometry

The gas chromatography-mass spectrometry (GC–MS) analysis of purslane, radish, and rosemary oils is listed in Tables 1, 2, and 3. In this study, about 50 constituents were identified in purslane oil. Most of these constituents are fatty acids, esters, and different aromatic compounds. The chemical composition, retention time, and chemical formula are summarized in Table 1. Purslane oil contained a higher concentration of aromatic hydrocarbons and the major components were benzene (1-methyldecy) (5.63), benzene (1-methylundecyl) (5.28), and benzene (1-methyldodecyl) (3.79). Furthermore, the radish seed oil shows a significant abundance of fatty acids with a total of 20 substances. These compounds are listed in Table 2, and the most abundant compounds were 2-Decenal, (E)- (13.59), Undecane (9.52), Tridecane (8.34), and Decane (8.20). Finally, as represented in Table 3, the major components in rosemary essential oil were eucalyptol (26.25%) followed by d-limonene (12.11).

**Table 1:** The main chemical constituents of *Portulaca oleracea* (purslane) oil by GC–MS analysis

Retention time	Compound name	Area (%)	Molecular formula
9.21	3,5-Heptadienal	1.53	C_10_H_14_O
9.81	Bicyclo[3.3.1]non-6-en-2-ylamine	0.99	C_9_H_15_N
14.82	Benzene, (1-butylhexyl)-	1.65	C_16_H_26_
15.05	Benzene, (1-nitropropyl)-butane	1.14	C_9_H_11_NO_2_
15.48	Benzene, (1-butyloctyl)	2.18	C_18_H_30_
16.35	10-Chlorotricyclo[4.2.2.0(1,5)]dec-7-ene	2.73	C_10_H_13_Cl
16.99	Benzene, (1-pentylhexyl)	1.91	C_17_H_28_
17.07	Benzene, 1,2,4-tripropyl	3.52	C_15_H_24_
17.30	1H-Indole-2,3-dione, 7-propy	3.46	C_11_H_11_NO_2_
17.77	Benzene, (1-ethylnonyl)	3.63	C_17_H_28_
18.61	Benzene, (1-methyldecy)	5.63	C_17_H_28_
19.13	Benzene, (1-pentylheptyl	2.73	C_18_H_30_
19.22	Benzene, (1-butyloctyl)-	2.80	C_18_H_30_
19.49	Benzene, (1-propylnonyl)-	2.61	C_18_H_30_
19.95	Benzene, (1-ethyldecyl)-	3.44	C_18_H_30_
20.78	Benzene,(1-methylundecyl)-	5.28	C_18_H_30_
21.17	Benzene, (1-pentyloctyl)-	2.99	C_19_H_32_
21.31	Benzene, (1-propylnonyl)-	2.12	C_18_H_30_
1.57	Benzene, (1-propyldecyl)-	2.16	C_19_H_32_
22.04	Benzene,(1-ethylundecyl)-	2.20	C_19_H_32_
22.61	Bicyclo[3.3.1]non-3-en-2-ol, exo-	0.65	C_9_H_14_O
22.92	Benzene, (1-methyldodecyl)-	3.79	C_19_H_32_
23.40	Silicic acid, diethyl bis(trimethylsilyl) ester	0.90	C_10_H_28_O_4_Si_3_
24. 41	Pyrrolo[1,2-a]pyrazine-1, 4-dione,hexahydro-3-(2-methylpropyl)-	1.68	C_11_H_18_N_2_O_2_
26.62	6-Octadecenoic acid	0.93	C_18_H_34_O_2_
27.09	2-Cyclohexylpiperidine	0.77	C_11_H_21_N
29.48	Methyl3,4-tetradecadienoate	1.15	C_15_H_26_O_2_
29.66	1-Bromoeicosane	0.69	C_20_H_41_Br
30.31	1,5,9,11-Tridecatetraene,12-methyl-, (E,E)-	3.27	C_14_H_22_
30.50	(2,2,6-Trimethyl-bicyclo[4.1.0]hept-1-yl)-methanol	0. 60	C_11_H_20_O
30.75	1-Tri(isobutyl)silyloxytetrAdecane	0.63	C_26_H_56_OSi
31.12	Hexadecane, 1-chloro-	0.87	C_16_H_33_Cl
31.54	d-( +)-Glucuronic acid	0.70	C_6_H_10_O7
31.71	2-Pentene,3-(chloroethylboryl)−2-(chlorodimethylsilyl)-, (E)-	0.86	C_9_H_19_BCl_2_Si
31.80	9,12,15-Octadecatrienoic acid, 2-[(trimethylsilyl)oxy]−1-[ [(trimethylsilyl)oxy]methy l]ethyl ester, (Z,Z,Z)-	1.67	C_27_H_52_O_4_Si_2_
31.85	Methyl(Z)−5,11,14,17-eicosatetrae Noate	1.88	C_21_H_34_O_2_
32.07	4-Methyldocosane	2.33	C_23_H_48_
32.14	Eicosane, 3-methyl-	2.42	C_21_H_44_
32.42	tert-Hexadecanethiol	0.88	C_16_H_34_S
33.70	Octatriacontyl pentafluoropropionate	2.87	C_41_H_77_F_5_O_2_
33.86	1-Monolinoleoylglycerol trimethylsilyl ether	1.38	C_27_H_54_O_4_Si_2_
34.01	Spirost-8-en-11-one,3-hydroxy-, (3á,5à,14á,20á,22á,25R)-	1.83	C_27_H_40_O_4_
34.11	Glyceryl tridecanoate	1.96	C_33_H_62_O_6_
34.27	Androst-5-en-3-one,19-acetoxy-4,4-dimethyl-,Oxime	3.52	C_23_H_35_NO_3_
34.41	2-Oxazolamine, 4,5-dihydro-5-(phenoxymethyl)-N-phenyl-	0.80	C_16_H_16_N_2_O_2_
34.49	Gibb-3-ene-1,10-dicarboxylic acid,2,4a-dihydroxy-1-methyl-8-methylene-,1,4a-lactone, 10-methyl ester, (1à,2á,4aà,4bá,10á)-	1.25	C_20_H_24_O_5_
34.55	Pregnan-18-oic acid,20-hydroxy-, (5à)-	1.00	C_21_H_34_O_3_
34.99	Heptacosane	1.77	C_27_H_56_
35.21	Octasiloxane,1,1,3,3,5,5,7,7,9,9,11,11,13,13,15,15-hexadecamethyl-	0.76	C_16_H_50_O_7_Si_8_
35.71	ç-Sitosterol	1.50	C_29_H_50_O

**Table 2:** The main chemical constituents of *Raphanus sativus* (radish) oil by GC–MS analysis

Retention time	Compound name	Concentration	Molecular formula
5.09	3-Benzylsulfanyl-3-fluoro-2-trifluoromethyl-acrylic acid methyl ester	5.17	C_12_H_10_F_4_O_2_S
5.72	Octane	8.16	C_8_H_18_
9.19	Octadecane, 3-ethyl-5-(2-thylbutyl)	2.96	C_26_H_54_
10.64	Dodecanoic acid, tricosafluoro	2.36	C_12_HF_23_O_2_
11.32	Decane	8.20	C_10_H_22_
11.95	10,13-Octadecadiynoic acid, methyl ester	2.67	C_19_H_30_O_2_
12.34	Octadecane, 3-ethyl-5-(2-ethylbutyl)	2.85	C_26_H_54_
12.86	Undecane	9.52	C_11_H_24_
13.20	1,13-Tridecanediol, diacetate	3.62	C_17_H_32_O_4_
14.47	Undecane, 2,6-dimethyl-	8.34	C_13_H_28_
15.20	Pregna-1,4-dien-3-one, 11á,17,20à,21-tetrahydroxy-, cyclic 20,21-acetal with acetone	5.78	C_24_H_34_O_5_
16.08	2-Decenal, (E)-	13.59	C_10_H_18_O
16.85	2-Myristynoyl pantetheine	3.21	C_25_H_44_N_2_O_5_S
17.15	Ethanol, 2-(9,12-octadecadienylox y)-, (Z,Z)	4.23	C_20_H_38_O_2_
17.73	2-Dodecenal, (E)-	3.18	C_12_H_22_O
18.23	Hexadecanoic acid, 1(hydroxymethyl)1,2ethanediyl ester	3.35	C_35_H_68_O_5_
20.19	1-Eicosanol	2.40	C_20_H_42_O
21.12	Cyclohexane, 1,1′-dodecylidenebis[4-me thyl	2.61	C_26_H_50_
21.41	2-Propenoic acid, pentadecyl ester	4.63	C_18_H_34_O_2_
21.79	Methyl tetradecanoate	3.15	C_15_H_30_O_2_

**Table 3:** The main chemical constituents of *Rosmarinus officinalis* (rosemary) oil by GC–MS analysis

Retention time	Compound name	Concentration	Molecular formula
10.03	Cyclohexane, 1-methylene-4-(1-methylet henyl)	13.00	C_10_H_16_
10.37	á-Pinene	6.00	C_10_H_16_
10.97	4-Carene, (1S,3R,6R)-(-)	7.10	C_10_H_16_
11.93	d-Limonene	12.11	C_10_H_16_
11.99	Eucalyptol	26.25	C_10_H_18_O
12.99	1,5-Heptadiene, 2,6-dimethyl	1.04	C_9_H_16_
13.20	1,6-Octadien-3-ol, 3,7-dimethyl-, acetate	1.48	C_12_H_20_O_2_
13.94	Bicyclo[2.2.1]heptan-2-on e, 1,7,7-trimethyl-, (1S)	5.22	C_10_H_16_O
14.89	3-Cyclohexene-1-methano l, à,à4-trimethyl	2.18	C_10_H_18_O
15.19	Fenchyl acetate	1.93	C_12_H_20_O_2_
16.56	Acetic acid, 1,7,7-trimethyl-bicyclo[2.2 .1]hept-2-yl ester	6.89	C_12_H_20_O_2_
17.12	4,6-Octadienoic acid, 2-acetyl-2-methyl-, ethyl ester	1.19	C_13_H_20_O_3_
18.55	Bicyclo[7.2.0]undec-4-en e, 4,11,11-trimethyl-8-methy lene	2.01	C_15_H_24_
19.63	Hexadecanoic acid, methyl ester	10.09	C_17_H_34_O_2_
21.25	Eicosanoic acid	3.52	C_20_H_40_O_2_

### Characterization of *P. oleracea*,* R. sativus, *and *R. officinalis* Oil Nano-Emulsions

**Fig. 1:**
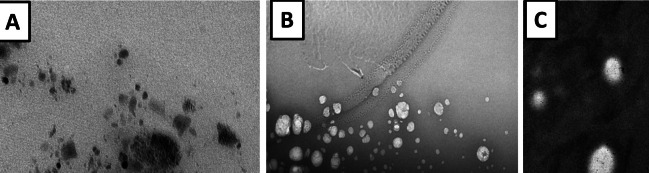
Transmission electron microscope (TEM) of the prepared oil nano-emulsions for *Portulaca oleracea* (**A**), *Raphanus sativus* (**B**), and *Rosmarinus officinalis* (**C**)

**Fig. 2:**
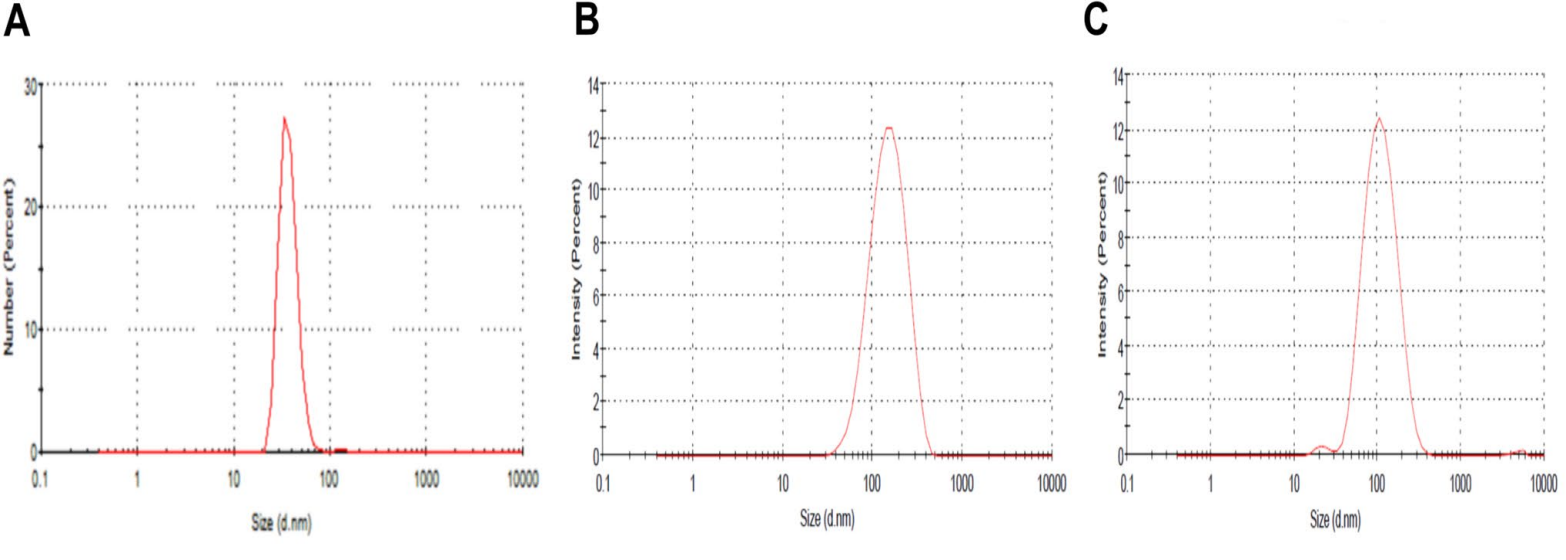
Dynamic light scattering (DLS) of the prepared oil nano-emulsions for *Portulaca oleracea* (**A**), *Raphanus sativus* (**B**), and *Rosmarinus officinalis* (**C**)

The prepared nano-emulsion formulations which contain 10% (ml/ml) of *P. oleracea*, *R. sativus*, and *R. officinalis* were characterized in terms of droplet size, polydispersity index (PDI), zeta potential, morphological structure, and thermodynamic characterization (Table 4). The droplet size distribution and morphological structure of purslane, rosemary, and radish oil nano-emulsions are shown in Figs. 1 and 2. In our study, the average size of the nano-emulsions was found to be less than 200 nm; the particle sizes for purslane radish and rosemary are 26.67 nm, 134.9 nm, and 97.36 nm. The obtained values in this study of PDI are less than 0.3; the PDI of purslane oil is 0.186, 0.190 for radish oil, and 0.202 for rosemary. Zeta potential which characterizes the surface charge of the nano-emulsion particles is an important factor for nano-emulsion stability. Zeta potentials of purslane, radish, and rosemary nano-emulsions are − 0.69 mV, − 29.9, mV, and − 18.00 mV, respectively (Table 4). The TEM images (Fig. 2) investigated the spherical appearance of synthesized o/w nano-emulsion and showed droplet mean size in the range of 200 nm.

**Table 4:** Characterization of *Portulaca oleracea*, *Raphanus sativus*, and *Rosmarinus officinalis* oil nano-emulsions

Nano-emulsion oil	Size-average (d.nm)	PDI	Zeta potential ± zeta deviation (mV)	Conductivity (mS/cm)	viscosity	Thermodynamic characterization		
						Freeze thaw cycle	Centrifugation	Heating–cooling cycle
*Portulaca oleracea*	26.67	0. 18	− 0.69	0.321	24.7	√	√	√
*Raphanus sativus*	134.90	0.19	− 29.9	0. 236	26	√	√	√
*Rosmarinus officinalis*	97.36	0.20	− 18.00	0.231	6.5	√	√	√

*PDI* poly disparity index

√—passed the test

The stability of nano-emulsion formulations is an important character that indicates the shelf-life of the formulations. The prepared nano-emulsions passed the thermodynamic tests (freeze–thaw cycle, centrifugation, and heating–cooling cycle). The nano-emulsions showed stable formulation without any sign of instability such as phase separation sedimentation or creaming. The nano-formulations were physically stable up to 3 months from the preparation. The viscosity of prepared nano-emulsions is 24.7 mPas for purslane, 26 mPas for radish, and 6.5 mPas for rosemary (Table 4).

### Toxicity Effects of Oils and Their Nano-Emulsions

The pesticidal efficacy of purslane, radish, and rosemary oil–based nano-emulsions was compared to that of the pure oil against *A. gossypii*, *S. littoralis*, and *T. urticae* in this study. It is clear from data presented in Tables 5 and 6 that all formulation forms caused different mortality levels against the selected pests after 24 h from exposure.

**Table 5:** Pesticidal effect of the tested oils and their nano-emulsions on *Aphis gossypii* and *Tetranychus uritica* under laboratory conditions

Compounds	LC_50_^a^ (mg/L)	Confidence limits		LC_90_^b^ (mg/L)	Confidence limits		Slope^c^ ± SE	Intercept^d^ ± SE	(*χ*^2^)^e^
		Lower	Upper		Lower	Upper			
*Aphis gossypii*									
Purslane oil	85.02	24.33	167.73	427.68	206.29	5469.43	1.83 ± 0.18	− 3.52 ± 0.38	4.85
Purslane oil nano-emulsion	72.74	16.76	197.37	4608.90	950.74	1,263,067.25	0.71 ± 0.09	− 1.32 ± 0.19	7.20
Radish oil	555.42	320.59	1383.91	69,163.13	9998.13	66,885,031.55	0.61 ± 0.18	− 1.68 ± 0.46	0.59
Radish oil nano − emulsion	453.91	303.19	729.28	14,417.18	4741.92	195,608.94	0.85 ± 0.18	− 2.27 ± 0.47	0.13
Rosemary oil	869.64	699.79	1165.82	4346.75	2684.07	9832.30	1.83 ± 0.26	− 5.39 ± 0.70	0.01
Rosemary oil nano-emulsion	72.45	2.23	165.76	22,429.24	3883.65	142,208,243.29	0.51 ± 0.18	− 0.96 ± 0.46	1.27
*Tetranychus uritica*									
Purslane oil	291.05	240.54	344.44	1099.51	899.88	1410.96	2.22 ± 0.18	− 5.47 ± 0.49	0.15
Purslane oil nano-emulsion	235.86	176.94	316.06	3795.84	2083.34	9717.97	1.06 ± 0.13	− 2.52 ± 0.31	0.17
Radish oil	359.95	251.31	439.51	868.28	750.27	1075.01	3.35 ± 0.58	− 8.57 ± 1.64	1.25
Radish oil nano-emulsion	263.54	127.32	390.81	1733.28	1350.15	2533.43	1.57 ± 0.26	− 3.79 ± 0.80	0.21
Rosemary oil	337.46	220.86	415.61	748.21	650.12	920.94	3.71 ± 0.73	− 9.37 ± 2.05	0.24
Rosemary oil nano-emulsion	240.98	110.81	363.67	1507.04	1183.11	2141.39	1.61 ± 0.28	− 3.83 ± 0.84	2.74

^a^LC_50_ median lethal concentration (concentration which caused 50% mortality of the tested adults)

^b^Slope of the concentration—mortality regression line ± standard error

^c^LC_90_ concentration causing 90% death for the tested adults

^d^Intercept of the regression line ± S.E

^e^Chi-square value

**Table 6:** Insecticidal effect of tested oils and their nano-emulsions on the 4th larval instar of *Spodoptera littoralis* under laboratory conditions

Compounds	LD_50_^a^ (ppm)	Confidence limits		LD_90_^b^(ppm)	Confidence limits		Slope^c^ ± SE	Intercept^d^ ± SE	(*χ*^2^)^e^
		Lower	Upper		Lower	Upper			
Purslane oil	90.61	7.78	11.77	520.29	330.01	1300.89	1.74 ± 0.31	− 1.71 ± 0.32	0.29
Purslane oil nano-emulsion	55.35	45.08	64.19	154.64	129.51	202.08	2.87 ± 0.38	− 5.00 ± 0.73	0.91
Radish oil	140.89	10.68	31.05	2820.38	800.17	33,907.76	1.00 ± 0.30	− 1.18 ± 0.31	0.78
Radish oil nano-emulsion	123.06	113.12	134.31	221.39	195.29	262.38	5.02 ± 0.47	− 10.50 ± 0.98	0.71
Rosemary oil	120.07	10.73	13.75	310.43	250.13	430.95	3.08 ± 0.35	− 3.34 ± 0.37	0.81
Rosemary oil nano-emulsion	113.05	102.15	125.72	247.97	209.76	313.79	3.76 ± 0.38	− 7.71 ± 0.77	0.32

^a^LD_50_ median lethal dose (dose which caused 50% mortality of the treated larvae )

^b^Slope of the concentration—mortality regression line ± standard error

^c^LD_90_ dose causing 90% death for the treated larvae

^d^Intercept of the regression line ± S.E

^e^Chi-square value

The results showed that the tested oils have positive toxic effects, and purslane oil exhibited the highest insecticidal activity against *A. gossypii* with LC_50_ = 85.02 mg/L followed by radish and rosemary with LC_50_ = 555.42 and 869.64 mg/L, respectively. However, rosemary and purslane oil nano-emulsion record the same greatest impact on *A. gossypii* with LC_50_ = 72.45 and 72.74 mg/L, respectively, followed in descending order with radish oil nano-emulsion with LC_50_ = 453.91 mg/L. Furthermore, the acaricidal activity of the tested oils and their nano-emulsion against *T. urticae* is presented in Table 5. According to the LC_50_ values, purslane oil is most active than radish and rosemary oil, with LC_50_ values = (291.05, 235.86), (359.95, 263.54) and (337.46, 240.98) mg/L, for bulk and nano-emulsion oil, respectively. These results indicate that the tested oil nano-emulsions have efficiency and may be used as insecticide and acaricide agents.

The insecticidal effects of the tested oils and their nano-emulsions using the residual bioassay method on 4th instar larvae of *S. littoralis* are shown as LD_50_ values in Table 6. All oils caused mortality against *S. littoralis* compared with the control, the result indicating that among the tested oils, purslane oil (nano and bulk) had a high toxic effect against *S. littoralis* after 24 h (LD_50_ = 55.35 ppm and 96.1 ppm), followed by rosemary (nano and bulk) with LD_50_ = 120 ppm and 113 ppm, respectively. Conversely, radish oil (nano and bulk) was less effective on *S. littoralis* with LD_50_ = 123.06 and 140.89 ppm (nano and bulk).

## Discussion

Plant oils contain many active eco-friendly components with insecticidal properties; they may offer possible options to be used as insect control agents because of the low toxicity against human and animals, the environment, and non-target organisms (Khursheed et al. 2022). The insecticidal activity of oils may be due to the presence and variety of the active components (Khan et al. 2023). However, the chemical composition of oils can vary according to many factors; for instance, Abbasi et al. (2008) found that the total phenolic content of the pomegranate seed oil extracted by supercritical fluid extraction was many times higher than that oils extracted by organic solvents. In addition, the stage of plant growth can influence the oil’s composition; Sefidkon et al. (2007) extracted essential oil from *Satureja rechingeri* and found that carvacrol (the main essential oil component) percentage was 56.1% at the beginning of flowering and 84.0–89.3% at the full flowering stage. Additionally, different fertilizer rate can have an impact on the composition of essential oils (Nurzyńska-Wierdak 2013).

GC/MS was used to recognize the components of the tested oils. Radish oil is found to be rich in fatty acids and hydrocarbons. The investigations are in agreement with those reported in other studies conducted by Selyutina and Gapontseva (2016) and Ibrahim et al. (2020) who isolate various components from different varieties of radish which were hexadecanoic acid, thiocyanate compounds, octadecanoic acid, sulfur compounds, thiirane butanenitrile, l-glutamic acid, hydrocinnamic acid nonanal, pentadekanal, dioktylftalat, dyizobutylftalat squalene, linoleic acid, oleic acid, stearic acid, trycosane, tetracosane, pentacosane, pyrrolidine, hexacosane caryophyllene oxide, methyl oleate, and heptacosane. In addition, Waheed et al. (2019) and Ibrahim et al. (2020) found that radish oil is rich in fatty acids, hydrocarbons, and sulfur compounds (oleic acid, n-hexadecanoic acid, oleic acid octadecanoic acid, and erucic acid).

Many previous studies (Tucker and Maciarello 1986; Boutekedjiret et al. 2003; Isman et al. 2008) analyzed rosemary essential oil by GC/MS and the result is in agreement with ours. The components of rosemary essential oil analyzed by Shokri et al. (2017) were 1.8-cineol (15.96%), α-pinene (13.38%), camphor (7.87%), bornyl acetate (6.54%), verbenone (5.82%), borneol (5.23%), camphene (4.96%), and (E)-caryophyllene (3.8%). Further, Zheljazkov et al. (2015) found that α-pinene, camphor, and eucalyptol were the major components of rosemary essential oil; Mossa et al. (2019) demonstrated that rosemary oil contained α-pinene (10.91%), d-limonene (9.19%). Eucalyptol, camphor, triacetin, 2*β-*pinene, and camphene were reported as the major active components (Ali et al. 2022). In general, the main composition of rosemary essential oil is 1,8 cineol, camphor, camphene, and α-pinene, in addition to borneol, limonene, myrcene, eucalyptol, and α-terpineol.

In our study, the average size of the prepared nano-emulsions was found to be less than 200 nm, which means that our formulations are within the standard size of nano-emulsions according to Shah et al. (2010) who clarified that nano-emulsions droplet size typically fall within range of 20 to 200 nm. The polydispersity index (PDI) which is an important parameter of nano-emulsion characteristics plays an important role in nano-formulation stability and release (Tamjidi et al. 2013). The values of PDI range from 0.0 to 1.0; values equal to or less than 0.3 are the most acceptable for the homogeneity of the droplet size in the formulations and reflect the quality concerning the formulation size distribution (Baboota et al. 2007; Clarke 2013). Zeta potential characterizes the surface charge of the nano-emulsion particles which are responsible for the repulsive force among nanoparticles; furthermore, it is an important factor for nano-emulsion stability. In general, Zeta potential value ± 30 mV usually reflects good stability of the colloid dispersion (Freitas and Müller 1998; Zhou et al. 2010).

In recent years, nano-formulations have been more potent than their bulk in terms of stability, volatility, and water miscibility (Mondal et al. 2022). Hence, our study clarified that nano-emulsions were effective in controlling the pests and these results are in agreement with many research. Abdel‐Raheem et al. (2020) confirmed that purslane oil (bulk and nano) showed a good impact on *R. ferrugineus* larvae and adults with a mortality percentage of 67.2% and 83.5% for adults and 75.2% and 92.5% for larvae. Moreover, Sabbour and Abd El-Aziz (2017) conducted a study demonstrating that both purslane oil and its nano-emulsion significantly reduced the emergence rate of *E. kuehniella* moths; this suggests that both forms of the oil hold promise as a non-chemical method for controlling moth populations. Aly et al. (2022) explored the potential of *P. oleracea* extract as a natural pesticide against the aphid *Aphis craccivora*, utilizing slide dipping and spraying techniques under laboratory conditions. They achieved a remarkable EC_50_ value of 0.321 ppm for both methods and highlighting the extract’s efficacy as a promising eco-friendly alternative to conventional insecticides. Further, Wang et al. (2020) identified specific compounds in purslane (homo iso-flavonoids and aliphatic acids) that effectively inhibited and caused structural damage to the mid-gut of the *Spodoptera litura* (Fab.) larvae. This aligns with the findings of Zhongning (2009), who demonstrated that petroleum ether extracts from purslane exhibited strong contact and antifeeding toxicity against *Plutella xylostella* (L.) larvae with significant mortality rates observed at high concentrations.

On the other hand, radish oil exhibited insecticidal activity against many pests. The oil illustrates insecticidal effects against various insects such as *A. gossypii* (Ibrahim et al. 2020), *Aphis fabae* and *Macrosiphum rosae* (Alghamdi 2018), *Lucilia sericata* (Khater and Khater 2009), and *Phenacoccus solenopsis* (Abdel-Mogib 2019), in addition to its antibacterial activity (Khan et al. 2018) and antifungal activity (Elshaer et al. 2019). Furthermore, radish seed oil caused significant mortality against *A. fabae* and *M. rosae* with 82.5% and 69.2%, respectively (Alghamdi 2018). Our results agreed with Ibrahim et al. (2020) who studied the toxicity of different extractions of *R. sativus* roots against adults and second-instar nymphs of *A. gossypii* under laboratory conditions and they found that the LC_50_ values were in the range of 309.43:636.2 ppm against adults and nymphs, respectively. Moreover, radish demonstrate significant effects on *S. littoralis*; Al-Shannaf (2011) compared the feeding behavior of *S. littoralis* larvae on radish and tomato leaves. Larvae which fed on radish leaves consumed significantly less food with an average intake of 1.43 g and 1.15 g in the first and second generations, respectively. In contrast, larvae which fed on tomato leaves consumed much higher amounts with an average consumption of 6.13 g and 6.20 g in the first and second generations, respectively.

Another research demonstrated the potential activity of radish oil conducted by Khater and Khater (2009) who studied the effect of the oral treatment with 6.93% radish oil against *Lucilia sericata* larvae and the treatment achieved 50% mortality rate (LC_50_) in 3rd instar larvae, while a higher concentration of 12% oil completely stopped adult fly emergence. According to our knowledge, the preparation and testing of radish oil nano-emulsions against insects is an entirely novel area of research with the potential to offer a unique and eco-friendly approach to pest control.

Rosemary oil is an active biopesticide against a range of pests such as stored grain insects (Hanane et al. 2018; Teke and Mutlu 2021), larvae (Tak and Isman 2015), mites (Miresmailli and Isman 2006), and aphids (Sayed et al. 2022), in addition to its antibacterial and anti-fungal activity (Bozin et al. 2007). Our results demonstrated that rosemary essential oil and its nano-formulatin can induce toxicity on *A. gossypii*, *S. littoralis*, and *T. urticae* and these results are in agreement with many authors. Santana-Méridas et al. (2014) found that the ethanolic extract of rosemary had high antifeedant activity against *S. littoralis* and *M. persicae*. Tortorici et al. (2022) found that nanostructured lipid carriers of rosemary reduced the feeding activity of *S. littoralis*. In the same context, Joo and Hussein (2023) found that rosemary oil has insecticidal activity against the fall armyworm *Spodoptera frugiperda* under laboratory conditions with 100% mortality at 0.2% concentration. Mossa et al. (2019) prepared nano-sized rosemary essential oil and studied its effect on *T. urticae* by spray technique; they found that the LC_50_ of the bulk oil was 1578.50 and 1.829 μg/ml^−1^ for immature and adult females of spider mites, respectively. However, the LC_50_ of nano rosemary was 723.71 and 865.68 μg/ml^−1^ for immature and adult females, respectively. A similar result was achieved by Tortorici et al. (2022), who tested rosemary as nanostructured lipid carriers against *A. gossypii* and found that rosemary nanostructured caused a high mortality percentage and affected progeny by topical contact treatment.

Our study significantly contributes to the field by confirming the effectiveness of oil nano-emulsions as novel delivery tools for harnessing the bio-pesticidal power of plant oils. This aligns with prior research on the superior insecticidal activity of nano-formulations compared to their bulk counterparts, highlighting the exciting potential of this technology for developing sustainable pest management solutions (Badawy et al. 2018; Mossa et al. 2019; Elnabi et al. 2021; Spinozzi et al. 2021; Metwally et al. 2022; Nenaah et al. 2022; Salazar et al. 2022).

The results and insights gained from this study may highlight the promising potential of nano-emulsion-based plant oils as the next generation of safe nano-pesticides. Thus, for better applications, more research is needed to investigate the efficacy of oil nano-emulsions in field. In addition, it is essential to evaluate the stability of pesticide nano-emulsions under different conditions, such as pH levels and temperature. Additionally, it is important to study the cytotoxic and genotoxic effects of oil nano-emulsions on animal cells.

## Conclusion

The results of this study confirm that nano-emulsions can effectively deliver plant oils in stable and homogeneous nanoparticles. This presents an exciting opportunity to use natural bioactive plant compounds as sustainable and effective bio-pesticides against harmful pests. The nano-emulsion formulations of *P. oleracea*, *R. sativus*, and *R. officinalis* oils demonstrated insecticidal activity against *A. gossypii*, *T. urticae*, and *S. littoralis* and showed improved effectiveness compared to the use of the pure oils. GC–MS analysis of purslane seed oil, radish seed oil, and rosemary essential oil revealed the presence of various constituents, primarily fatty acids, phenols, terpenes, esters, and other aromatic compounds. Among the tested oils, purslane oil nano-emulsion proved to be the most potent insecticide, followed by rosemary and radish oils. This suggests that nano-emulsions containing these plant oils hold significant potential as natural alternatives to synthetic pesticides within Integrated Pest Management (IPM) programs. For commercialization of these novel nano-emulsions, successful field trials and additional biosafety evaluations of the formulations under study are necessary.
